# Revealing the Immune Infiltration Landscape and Identifying Diagnostic Biomarkers for Lumbar Disc Herniation

**DOI:** 10.3389/fimmu.2021.666355

**Published:** 2021-05-27

**Authors:** Linbang Wang, Tao He, Jingkun Liu, Jiaojiao Tai, Bing Wang, Lanyue Zhang, Zhengxue Quan

**Affiliations:** ^1^ Department of Orthopedic Surgery, The First Affiliated Hospital of Chongqing Medical University, Chongqing, China; ^2^ Honghui Hospital, Xi'an Jiaotong University, Xi'an, China; ^3^ Laboratory of Environmental Monitoring, Shaanxi Province Health Inspection Institution, Xi'an, China; ^4^ Traditional Chinese Medicine Department, Chongqing Medical University, Chongqing, China

**Keywords:** intervertebral disc degeneration, lumbar disc herniation, macrophages, nucleus pulposus, single-cell sequencing (SCS)

## Abstract

Intervertebral disc (IVD) degeneration and its inflammatory microenvironment ultimately led to discogenic pain, which is thought to originate in the nucleus pulposus (NP). In this study, key genes involved in NP tissue immune infiltration in lumbar disc herniation (LDH) were identified by bioinformatic analysis. Gene expression profiles were downloaded from the Gene Expression Omnibus (GEO) database. The CIBERSORT algorithm was used to analyze the immune infiltration into NP tissue between the LDH and control groups. Hub genes were identified by the WGCNA R package in Bioconductor and single-cell sequencing data was analyzed using R packages. Gene expression levels were evaluated by quantitative real-time polymerase chain reaction. The immune infiltration profiles varied significantly between the LDH and control groups. Compared with control tissue, LDH tissue contained a higher proportion of regulatory T cells and macrophages, which are associated with the macrophage polarization process. The most significant module contained three hub genes and four subclusters of NP cells. Functional analysis of these genes was performed, the hub gene expression pattern was confirmed by PCR, and clinical features of the patients were investigated. Finally, we identified TGF-β and MAPK signaling pathways as crucial in this process and these pathways may provide diagnostic markers for LDH. We hypothesize that the hub genes expressed in the specific NP subclusters, along with the infiltrating macrophages play important roles in the pathogenesis of IVD degeneration and ultimately, disc herniation.

## Introduction

Low back pain is a widespread and complex clinical condition affecting 70%–85% of the population worldwide. However, in more than 40% of chronic lower back pain cases, there is no evidence of nerve compression (1). Therefore, intervertebral disc (IVD) degeneration is believed to be the main cause of pain (2).

The IVD has been identified as an immune privilege organ because its unique structure isolates the nucleus pulposus (NP) from the immune system of the host (3), in which the annulus fibrosus (AF), cartilaginous endplate, and immunosuppressive molecular factors consist of the blood-NP barrier (4). The NP triggers an immune response when the blood-NP barrier is damaged. This process plays a crucial role in IVD degeneration and leads to multiple pathological processes, in which the NP loses proteoglycans and becomes more fibrotic (5). Meanwhile, matrix metalloproteinases and inflammatory mediators, such as interleukin-1β (IL-1β) and tumor necrosis factor-alpha (TNF-α), are upregulated in the disc microenvironment. These cytokines are produced by IVD cells and immune cells, such as macrophages and CD8 T cells (6–8). However, the immune landscape and the role of epigenetic regulation in the pathological process of IVD degeneration remains unclear.

In this study, we first determined the specific types of immune cells that are involved in IVD degeneration by using gene expression matrices. Then, the hub genes involved in this process were screened. Single-cell analysis uncovered the expression patterns of hub genes in NP cell clusters, and the prospective clinical experiment confirmed the prognostic value of hub gene expression.

## Materials and Methods

### Data Collection and Preprocessing

The microarray data and corresponding clinical information, including 30 human disc tissues, were downloaded and filtered from the GEO dataset (GSE124272, GSE147383, and GSE153761). The single-cell transcriptome data from intervertebral discs were obtained from GEO (GSE154884). Multiple datasets of gene expression matrices were merged and the inter-batch differences were removed for further processing.

### Differentially Expressed Genes (DEGs)

The edgeR package was used to screen for DEGs in stage IV lumbar disc herniation (LDH) tissues and controls, including spondylolisthesis and LDH stages I to III. The selection criteria were |log2 FC| > 1.5, and false discovery rate (FDR) < 0.05. The volcano plot and heatmap of DEGs were generated by the pheatmap R package.

### Immune Cell Infiltration Estimation

The CIBERSORT deconvolution algorithm was applied to quantify the degree of infiltration of 22 types of immune cells through the transcriptome data. Investigated immune cells included plasma cells, resting memory CD4+ T cells, CD8+ T cells, naive CD4+ T cells, T follicular helper cells, regulatory T cells (Tregs), activated memory CD4+ T cells, gamma delta T cells, naive B cells, memory B cells, monocytes, M0 macrophages, M1 macrophages, M2 macrophages, resting natural killer (NK) cells, activated NK cells, activated mast cells, eosinophils, neutrophils, resting dendritic cells, activated dendritic cells, and resting mast cells. The differences between the two groups were compared using the wilcox test and the results were visualized by applying the vioplot package. Finally, the correlation between infiltration rate of each types of immune cells was determined by the corrplot package.

### Identification of Immune Cell Infiltration-Related Genes 

Immune cell infiltration-related genes were identified using the WGCNA R package. First, the gene expression matrices outliers were filtered by hierarchical cluster analysis. Then, the correlation coefficient of genes was constructed and transformed into a weighted adjacency matrix. Next, these genes were allocated into minimum-sized modules and a cluster dendrogram was drawn, and then merged with a height cutoff (cutoff < 0.3). The correlation between gene expression and sample trait (immune cell infiltration score) was determined by the criterion of gene significance (GS) > 0.5 and module membership (MM) > 0.8. The relevant genes in the module were then tested for correlation with all other genes, the screening conditions were set at cor > 0.7 and p < 0.01, the regulatory network was visualized using cytoscape. Finally, matascape was used to conducted functional analysis to the selected genes.

### Single-Cell Analysis

Single-cell RNA sequencing data sets from IVD tissue (GSE154884) were obtained. The Seurat pipeline was used for data reprocessing and to classify the cell groups, SingleR identified the cell type, and Monocle was used to analyze the cell differentiation trajectory.

### Functional Correlation Analysis

The clusterProfiler package (9) was used for performing gene ontology (GO) enrichment analyses on cell cluster marker genes, where p < 0.05 and FDR < 0.25 were considered significantly enriched.

### Patients

Nine patients who underwent posterior open discectomy for radiating pain due to single-level LDH (classified as Pfirrmann grade IV by MRI) were included. Six patients were female and three were male (10). The mean duration between onset of symptoms and operation was 13.5 weeks. Patients with degenerative spondylolisthesis or a history of diabetes mellitus or renal disease were excluded. As a control, 14 patients, including 8 females and 6 males, who underwent posterior open discectomy for neurological symptoms due to single-level lumbar disc herniation (classified as Pfirrmann grades I to III by MRI) were selected for this study. Patient immune responses were evaluated as either “high” or “low” and the results are shown in **Table 1**, to be specific, the immune status of each individual is evaluated by whether the percentage of total lymphocytes higher than the median level of 32 or the percentage of CD4-CD8- lymphocytes is lower than median level of 6. Tissues were excised and transferred to liquid nitrogen for RNA and protein extraction. Written informed consent was obtained from all patients. Clinical data were acquired from hospital records and pathology reports. The study protocol (approval number: 2020–171) was approved by The Ethics Committee of the Affiliated Hospital of Chongqing Medical University.

**Table 1:** Results of immune function quality of patients in different groups.

item	patient 1	patient 2	patient 3	patient 4	patient 5	patient 6	patient 7	patient 8	patient 9	patient 10	patient 11	patient 12	patient 13	patient 14	reference range
ZLBXB-FZ1	18.74	22.06	17.12	26.33	21.2	36.42	19.24	48.2	36.63	38.27	21.87	24.24	25.22	26.83	27.90~37.3
CD3+	66.23	85.43	84.9	78.27	73.18	69.48	53.91	62.94	67.12	82.01	72.32	76.81	74.29	74.21	26.00-76.80
CD3+CD4+CD8-	37.78	52.67	58.32	41.89	36.67	42.74	45.21	43.62	41.37	47.91	46.68	46.37	42.24	47.52	30-46
CD3+CD4-CD8+	28.42	27.69	27.12	34.31	34.13	24.31	16.33	26.25	23.9	27.12	23.88	26.16	27.22	24.89	19.2-33.6
CD4+CD8+	1.13	1.45	0.77	1.82	0.23	0.43	0.19	3.11	0.31	0.86	0.13	0.43	0.71	0.22	0-2.00
CD4-CD8-	6.42	5.36	3.78	3.26	10.17	4.12	2.45	1.72	1.23	3.90	2.91	1.99	2.03	2.46	0-12.00
CD3-CD19+	12.76	5.69	6.51	3.31	5.23	18.92	23.65	17.13	23.72	10.56	11.87	22.15	19.88	16.41	8.50-14.50
CD3-CD16/56+	14.91	112.13	10.98	16.89	22	15.12	11.17	16.83	9.92	8.23	10.56	11.77	6.93	8.59	9.50-23.50
CD3+CD16/56+	3.82	7.87	5.34	3.66	6.92	0.76	2.31	3.98	7.23	4.81	1.54	1.57	1.21	2.66	/
High or low flag	L	L	L	L	L	L	L	H	H	H	H	H	H	H	/

### Total Protein and RNA Extraction

IVD specimens obtained from patients were first homogenized in phosphate-buffered saline (Tissue Tearor kits, Racine, model 985–370). The supernatant was obtained by centrifugation at 15,000 rpm at 4°C for 30 min. The Bradford protein assay method (#500–0006; Biorad, Hercules, CA, USA) was used for protein quantification and the results were used for measurement of TNF-α and TGF-β. IVD specimens were also used for RNA extraction using the UNIQ-10 Column Total RNA Purification Kit (Sangon Biotech, China). The quality and concentration of RNA were evaluated using a SMA4000 microspectrophotometer (Merinton Instrument, Inc. MI, USA).

### ELISA

IVD extracts were quantitatively analyzed for TNF-α and TGF-β using an enzyme-linked immunosorbent assay (ELISA) kit (Beijing Jingmei Biological Engineering Co, Ltd, China) and measurement at 450 nm. TNF-α and TGF-β concentrations were extrapolated from the standard curve.

### Reverse Transcription and Quantitative Real-Time Polymerase Chain Reaction (qRT-PCR)

The RR047A cDNA synthesis kit (TaKaRa, China) was used to perform the reverse-transcription of the extracted RNA and the 2X SG Fast qPCR Master Mix (High Rox, B639273, BBI) was used for quantitative PCR of hub genes on an ABI PRISM 3700 instrument (Foster, CA, USA). GAPDH was used as an internal control and primers are as follows:

ID1-F: 5’ CTCAGCACCCTCAACGG 3’,
ID1-R: 5’ GATCGGTCTTGTTCTCCCTC 3’,
RAP2C-F: 5’ CCCTCCGTGCTGGAAATTCT 3’,
RAP2C-R: 5 ‘CCATGAAAGGACAGCCCCAT 3’,
PTPRK-F: 5’ ACAGAGTGGTGAAAATAGCAGGAA 3’,
PTPRK-R: 5’ TGACAACTAGGAGAAGGAGGATGA 3’,
GAPDH-F: 5’ TGGGTGTGAACCATGAGAAGT 3’, and
GAPDH-R: 5’ TGAGTCCTTCCACGATACCAA 3’.

## Results

### Research Design Summary

**Figure 1:**
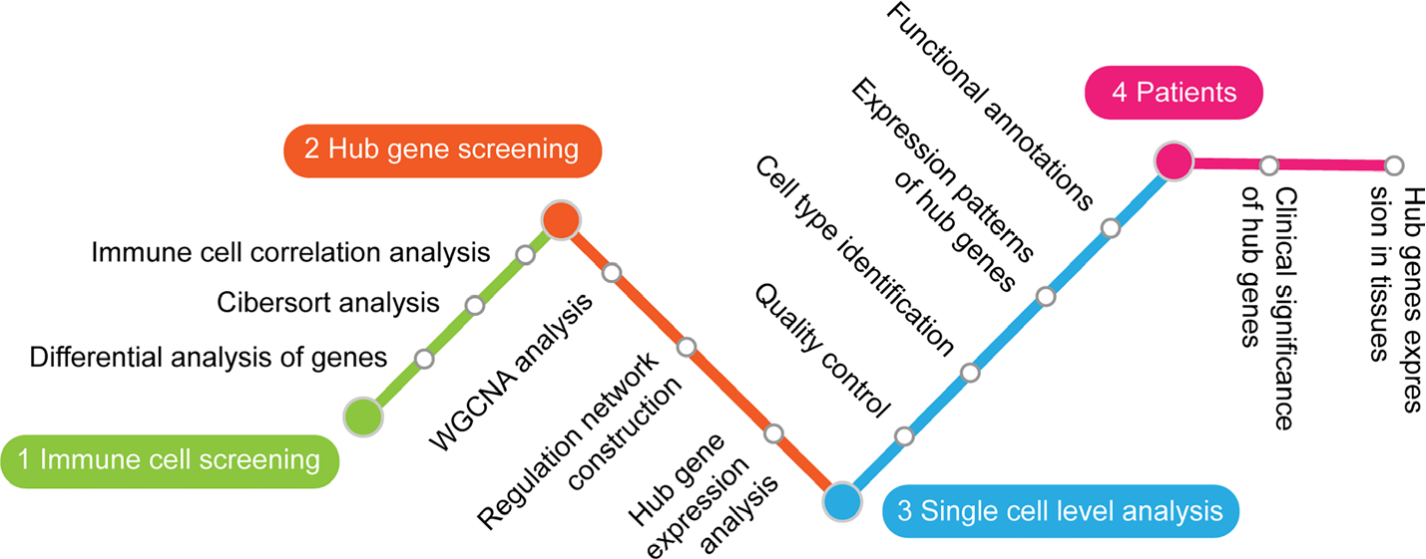
A flowchart showing the steps in this study.

A flow diagram showing the research design is shown in **Figure 1**. In brief, DEGs in the LDH were screened from microarray data of samples in the GEO database. CIBERSORT was then applied to DEGs for identifying LDH-related immune cells. Next, WGCNA and correlation test were used to find hub genes associated with the identified immune cells. The expression pattern of these hub genes was then examined at the single-cell level and several LDH-related NP cells were recognized. Finally, qRT-PCR and ELISAs were performed to verify the relationship between hub gene expression levels and clinical characteristics in LDH patients.

### Screening of DEGs

**Figure 2:**
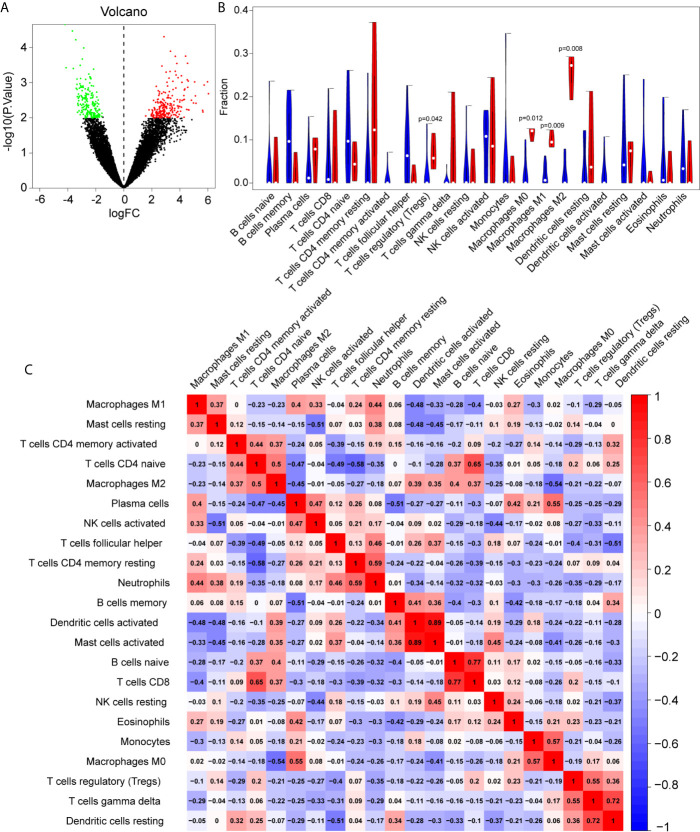
The differentially expressed genes (DEGs) and immune infiltration landscape in nucleus pulposus (NP) tissue. **(A)** Volcano map of DEGs, where red represents upregulated genes and green represents downregulated genes. **(B)** Immune infiltration differences between intervertebral disc grade IV degenerative tissue and control tissue. **(C)** Correlation matrix of 22 immune cell type proportions.

Batch correction, normalization, and difference analysis of RNA-seq data from GSE124272, GSE147383, and GSE153761 were performed to screen for DEGs in IVD samples. A total of 410 DEGs, including 195 downregulated and 215 upregulated genes were identified. The results were visualized using a volcano plot (**Figure 2A**), which identifies important genes.

### Immune Microenvironment Characteristics of Degenerated IVDs

In order to further reveal the immune microenvironment in degenerated IVDs, the CIBERSORT method was used to analyze specific immune cell types that infiltrated into IVD tissue. Among the 22 types of immune cells investigated, the results showed that Tregs and macrophage levels were significantly higher in degenerated IVD (p<0.05) (**Figure 2B**), As is shown in **Figure 2C**, by further analyzing the CIBERSORT scores, there was a positive strong correlation between Mast cells, neutrophils and M1 macrophages, On the other hand, there was a negative correlation between the infiltration of plasma cells, regulatory T cells and M1 macrophages.

### Identification of Immune Cell-Related Genes

**Figure 3:**
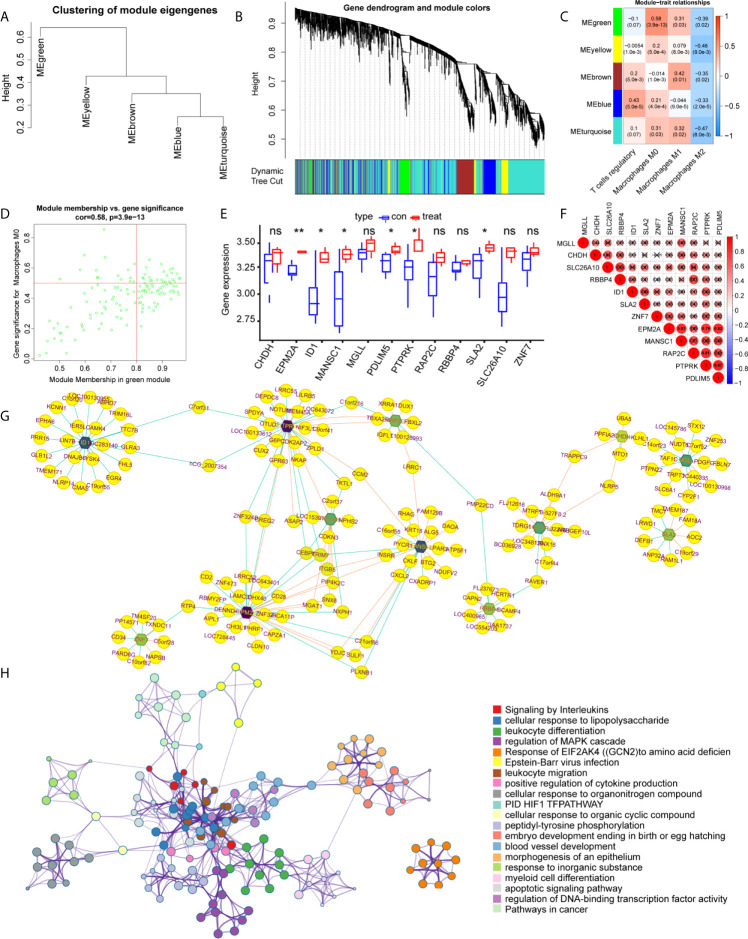
Identification of the key modules and genes that relate to immune infiltration features in intervertebral disc degeneration. **(A, B)** The construction of the co-expression network module clustering tree based on the 1-tom matrix. **(C)** Feature of each merged module relationship, with different colors representing different modules. Each row corresponds to a module and columns represent their correlations with the traits. Each unit includes the correlation coefficient and p-value. **(D)** Genes of the selected green module. **(E)** The hub gene expression levels in different groups. **(F)** Expression correlation matrix of each hub gene in lumbar disc herniation. **(G)** The gene regulation networks are constructed to identify the hub genes. The edge represents the protein binding conditions and the node represents each gene. Darkness of the node represents the edge number of each gene. **(H)** The functional enrichment of the correlated genes by using metascape. Symbol "*", "**", and "ns" respectively stand for P value under 0.05, P value under 0.01, and non-significance.

WGCNA was applied to identify differentially expressed immune cell-related genes from 410 DEGs, as shown in **Figures 3A–D**. These DEGs were then divided into modules and merged with different colors (**Figures 3A, B**). Five merged modules were analyzed and three gene modules were significantly correlated with immune cells (**Figure 3C**). Among them, we selected the green module, which was the most significant module that was related to macrophages M0, with a positive correlation of 0.58 and p<0.001 (**Figure 3D**). 15 genes were screened according to the criteria, of which 12 were selected by correlation test. The expression pattern of these key genes was then analyzed accordingly (**Figures 3E, F**). Cytoscape constructed the interaction network between these 12 genes and their target genes (**Figure 3G**). Next, to acquire more information about these genes we performed functional analysis using Matascape, as shown in **Figure 3H**, genes were enriched in critical biological processes, such as interleukin signaling and the regulation of cytokine production.

### scRNA-Seq Data Revealed High Cell Heterogeneity in IVD Tissue

**Figure 4:**
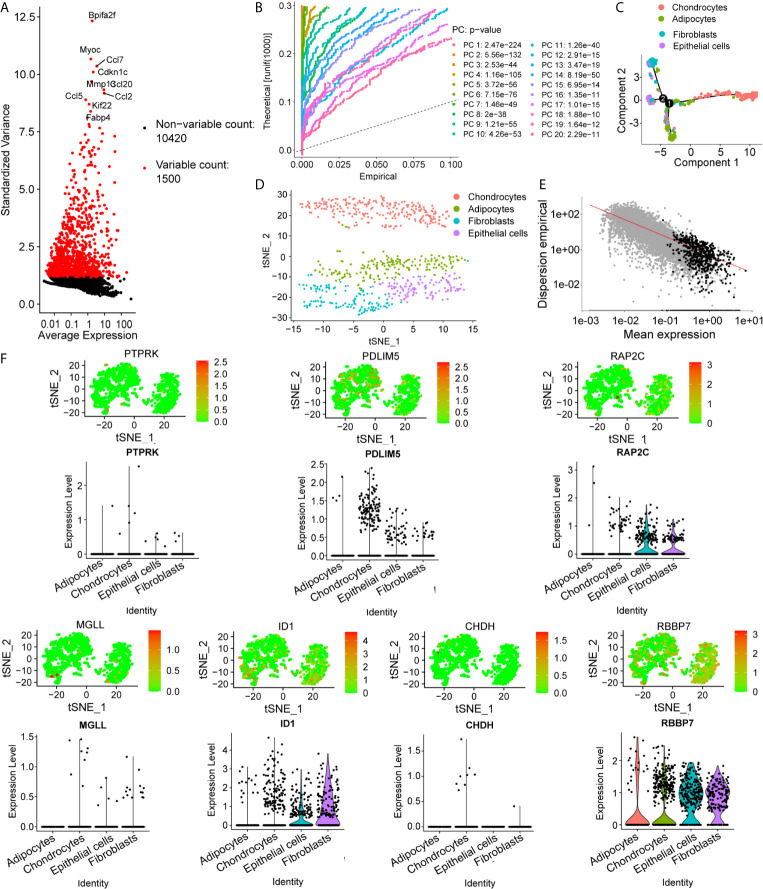
Preprocessing of the single-cell sequencing data and cell cluster identification. **(A, B)** Gene filtering and PCA clustering of the gene expression matrix. **(C)** t-SNE projections and cell subset annotation of intervertebral disc (IVD) tissue. **(D)** Quality control process for the trajectory analysis using the DDRTree method. **(E)** Cell trajectory analysis of single cells in IVDs shows distinct lineage cells in different clusters. **(F)** Expression pattern of key genes at the single-cell level, shown in t-SNE figures and violin maps.

To determine the single-cell level transcriptomic landscape of IVD compartments, we employed scRNA-sequencing data from healthy, non-degenerated rat IVDs. We first conducted quality control of the gene expression matrix (**Figure 4A**). Then, normalization of scRNA-seq data was performed and 20 principal components (p<0.05) were screened for subsequent analysis (**Figure 4B**). Reduced dimension process analysis was achieved by using Discriminative Dimensionality Reduction Tree (**Figure 4E**). Unsupervised analysis was then conducted for cell clustering using the t-distributed stochastic neighbor embedding (t-SNE) method (**Figures 4C, D**). The result showed high cell heterogeneity, in which IVD cells were segregated into four major distinct clusters, including chondrocytes (NP cells), fibroblasts (AF cells), adipocytes, and epithelial cells, which were determined using singleR and cell markers. Next, we tested the expression pattern of the hub genes in these cell clusters. As expected, most of the key genes were highly expressed in NP cells, with the key gene *ID1* found to be highly expressed in the AF cells (**Figure 4F**).

### Identification of NP Cell Clusters in IVD Tissue

**Figure 5:**
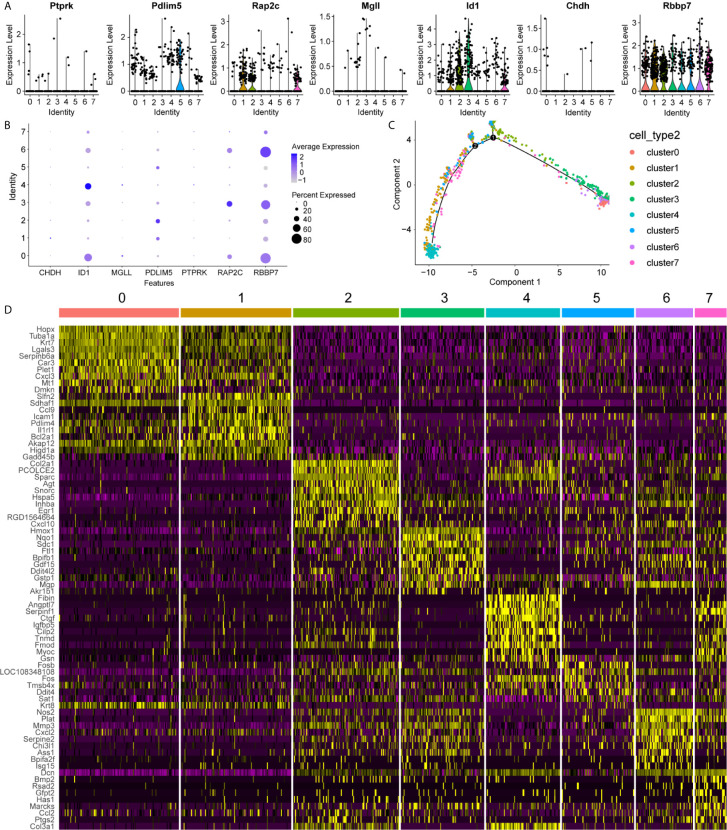
Clustering of nucleus pulposus (NP) cells, revealing key gene expression patterns. **(A, B)** The expression pattern of key genes in each NP cluster is shown in the violin maps and dot plot. Clusters 1, 2, 4, and 5 refer to each NP subcluster. In the dot plot, the average expression of each cluster is represented by a color gradient, in which lower expression is represented by a lighter color and higher expression is represented by darker color. The percentage of cells is indicated by dot size. **(C)** Cell trajectory analysis of the clusters, including NP cells. Noted that NP cells in cluster 5 are mainly located in the root, while other cells in clusters are distributed in branches. **(D)** The heatmap shows the marker genes in each cluster.

**Figure 6:**
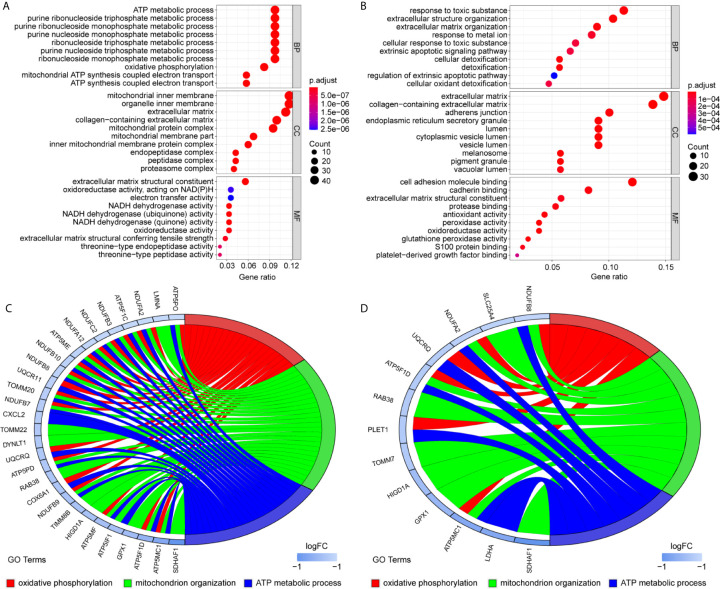
Gene markers and functional analysis of each nucleus pulposus (NP) cell cluster. Bubble plots showing GO/pathway analysis results of marker genes in NP cell cluster 1 **(A)** and cluster 2 **(B)**. The chord plots reveal highly relevant functions and genes assigned to NP cell cluster 1 **(C)** and cluster 2 **(D)**.

**Figure 7:**
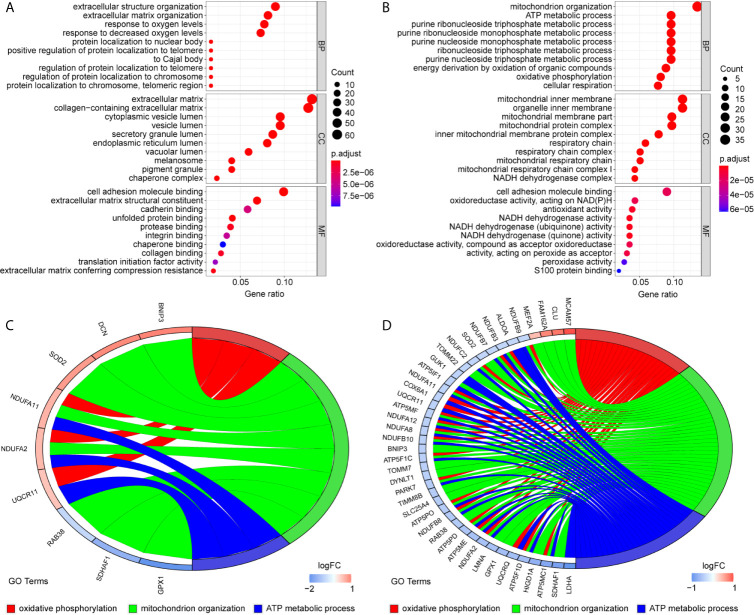
Gene markers and functional analysis of each nucleus pulposus (NP) cell cluster. Bubble plots showing GO/pathway analysis results of marker genes in NP cell cluster 4 **(A)** and cluster 5 **(B)**. The chord plots reveal highly relevant functions and genes assigned to NP cell cluster 4 **(C)** and cluster 5 **(D)**.

To uncover the detailed heterogeneity of NP cells, we re-subclustered the scRNA-seq data by upregulating the resolution value. Four cell clusters, including clusters 1, 2, 4, and 5, were identified according to their gene expression pattern (**Figure 5A**). Trajectory analysis was then performed to illustrate the degree of cell differentiation. All cells were projected onto one root and two branches. Interestingly, cells in cluster 5 were mainly located in the root, whereas cells in clusters 1, 4 were mostly located in the left, and most cells in cluster 2 were located in the right (**Figure 5C**). Based on the hub gene markers, clusters 1, 2, 4, and 5 were defined as RBB7+Pdlim5-, RBB7+ Pdlim5+, RBB7- ID1+, and RBB7- Pdlim5+ cell groups (**Figure 5B**). Next, we defined the molecular features of these NP cell groups by tracking their gene markers (**Figure 5D**). Among them, *TOMM20*, *TOMM22*, and *CXCL2* were recognized as specific marker genes in cluster 1 (**Figures 6A–C**). *TOMM20* has been shown to induce tissue inflammation responses in adipose and muscle tissue (11). *CXCL2* is reported to promote tumor cell migration through the induction of M2-like macrophage polarization. Therefore, we named cluster 1 “NP inflammatory response cells”. Genes, including *TOMM7* and placenta expressed transcript 1 (*PLET1*), are specifically expressed in cluster 2. *PLET1* encodes for a cell surface protein that is specifically expressed in trophoblast stem cells. Of interest, functions enriched in cluster 2 included regulation of extrinsic apoptotic pathway and responses to metal ions, suggesting that cluster 2 cells are involved in multidirectional maintenance and differentiation of chondroid cells (**Figures 6B–D**). Therefore, cluster 2 was named “NP repair cells”. *BNIP3*, *SOD2*, and other genes are highly expressed in cluster 4. Functional enrichment results from cluster 4 marker genes showed that these cells function to response to oxygen levels. Cluster 4 cells provide extracellular matrix structural constituents, which confer resistance to compression (**Figures 7A, C**). Thus, we named cluster 4 “extracellular matrix NP cells”. Cluster 5 cells have highly expressed genes, including *CLU*, *MEF2A*, *MCAM57*, and *FAM162A*, which are known markers of mesenchymal stem cells and NP progenitor cells (**Figures 7B–D**). The trajectory result showed that cluster 4 cells were mostly populated in the root of two branches. We speculate that this cell group of the IVD contains NP progenitor cells, which other researchers have suggested. Thus, we refer to this group as the “stem-like NP cells”.

### Hub Gene Expression Validation and Cytokine Levels in LDH Patients

**Figure 8:**
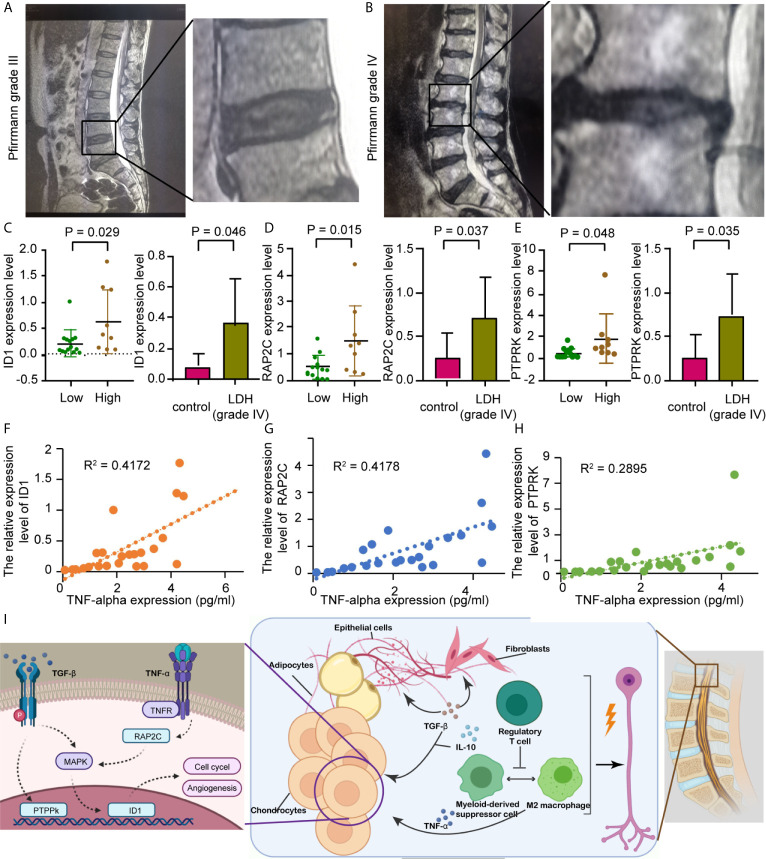
Prospective clinical experiment confirms the bioinformatic results. **(A, B)** The MRI image shows lumbar disc herniation in patients from different groups which are divided by Pfirrmann grades I-III or IV. **(C, E)** Comparison of preoperative hub gene expression levels. The box plots on the right show hub genes expression in in vertebral disc tissue from different groups, divided by the Pfirrmann disc grade; The left honeycomb map show hub genes expression levels in the high and low immunity groups, coffee color represent high immunity group and green represent low immunity group. **(C)** *ID1*, **(D)** *RAP2C*, **(E)** *PTPRK.* **(F)** Schematic showing that the extrusion of the nucleus pulposus in a degenerating intervertebral disc could cause multiple inflammatory responses, resulting in radicular pain. **(G–I)** Correlation between TNF-α level assessed by ELISA and hub genes expression, as previously described. R represents Pearson’s correlation coefficient. **(G)** *ID1*, **(H)** *RAP2C*, **(I)** *PTPRK.*

The Pfirrmann grade for each study group is represented by a set of lumbar intervertebral disc MRIs, as shown in **Figures 8A, B**. qRT-PCR results showed that the expression levels of ID1, RAP2C, and PTPRK were all significantly higher in NP tissues of the grade IV disc degeneration and high immunity groups (**Figures 8C–E**). ELISA results also showed that the expression of these genes was positively correlated with TNF-α (**Figures 8G–I**) but not with TGF-β (p<0.05, r=0.492). We believe that the expression of hub genes may play a role in chronic disorders such as IVD degeneration (**Figure 8F**).

## Discussion

IVD degeneration is one of the major contributors to radicular back and neck pain. However, structural degeneration of discs is not necessarily accompanied by pain (12). Therefore, the pain is likely a secondary event of leakage or injury to NP material through annular fissures caused by the recruitment of immune cells to the area with the structural deficit (13). IVD degeneration has been characterized by the infiltration of CD68 macrophages, T cells (CD4, CD8), and neutrophils in herniated discs. It is also accompanied by the appearance of invading blood vessels and nociceptive nerve fibers (14). NP, AF cells, and immune cells, such as macrophages, T cells, and neutrophils, have been reported to release cytokines like TNF-α, IL-1 α/β, IL-6, IL-17, IL-8, IL-2, IL-4, IL-10, and IFN-γ, as well as neurogenic factors, which promote discogenic pain and reinforce disc cell pathogenic processes, including senescence and autophagy (15).

In our study, infiltration of macrophages and Tregs was found to be increased in the degenerative IVDs compared with that in the control group. It is reported that the accumulation of macrophages is significantly higher with the progression of IVD degenerative grade. The presence of multiple macrophage markers, including CCR7+ and CD163+, is significantly higher in the NP, AF, and endplate regions of degenerative IVD with structural defects (16). Th17/Treg cells were reported to be involved in the pathogenesis of chronic low back pain through an immune response. The number of anti-inflammatory Tregs is higher in chronic lower back pain patients, along with alterations in the Th17/Treg ratio (17).

A great number of researches have demonstrated the involvement of aberrant epigenetic modification in many diseases, including Alzheimer’s disease and many other age-related diseases (18), as for the epigenetic modification in the IVD, we detected a relatively high expression of the inhibitor of DNA binding 1 protein (ID1) in both NP (mostly in cluster 4) and AF cells. ID1 is a nuclear protein that regulates cell growth by binding to DNA and preventing gene transcription. ID1 can inhibit the DNA binding and transcriptional activation ability of Helix-loop-helix (HLH) proteins with which it interacts. the latter of which are dimeric transcription factors that deposit or erase epigenetic marks, activate noncoding transcription, and sequester chromatin remodelers across the chromatin landscape. Its expression level is found to be correlated with multiple signaling pathways, including EGFR, K-Ras, MAPK, PI3K/Akt, and TGF-β, in various tumor types while facilitating angiogenesis (19). ID1 also plays an important role in tissue inflammation during orchestration (20). In fibroblasts, ID1 inhibits collagen expression through the TGF-β signaling pathway (21). ID1 production in rheumatoid arthritis synovial fibroblasts is mostly contained within exosomes, which could be affected by endothelial progenitor cells, leading to JNK signaling pathway activation in human dermal microvascular endothelial cells (20). We also noticed that IVD tissue expression of PTPRK is correlated with the pathological process of IVD degeneration. High PTPRK expression mediates homophilic intercellular interaction with adhesion junctions through its interaction with β and γ-catenin (22). RAP2C has also been reported to be involved in TNF-α-induced colorectal cancer metastasis (23). PCR results from IVD tissues showed that ID1, PTPRK, and RAP2C were highly expressed in stage IV tissues, which was consistent with our bioinformatics data. Our results also revealed that these genes were highly expressed in the high immune group. Combined with the results that hub gene expression was positively correlated with TNF secretion, we confirmed that with more IVD tissue degeneration, there was higher immune cell infiltration and higher hub gene expression, combining with previous studies and present results, we speculate that the pathological process of IVD degeneration has generated a special types of immune microenvironment that recruit regulatory T cells and multiple types of macrophages, the latter of which interact with NP cells, adipocytes, and fibroblasts through cytokines like TGF-β and IL-10, and brought abnormal gene expressions of ID1, PTPRK, and RAP2C in NP cells and promote the IVD pathological changes (**Figure 8I**).

The general cell components of the IVD includes cells of the NP and AF cells (24). However, the IVD cell type subclassification has been unrevealed. Despite similarities shared by these cells, there is high heterogeneity in terms of molecular phenotypic characteristics, extracellular matrix, and biomechanical behavior (25). In this study, we took advantage of single-cell sequencing technology to classify the IVD cells into four categories including NP, AF, adipocytes, and epithelial cells. The corresponding gene markers of each cluster were also elucidated, indicating new targets for the treatment of IVD-related disorders. Next, we performed a detailed cluster analysis for NP cells. NP cells were separated into two branches with distinct differentiation features, according to trajectory analysis. Four clusters of NP cells were identified and we speculated that the different roles for each cluster were influenced by the expression of certain genes. The cells in cluster 1 were inferred to be responsible for the inflammatory process through immune cells such as macrophages. The cells in cluster 4 are also worth investigating since their stem-like characteristics have great therapeutic potential (26). The marker genes in clusters 2 and 3 are mainly related to stress and fibrotic changes. The relationships between the various populations of NP and AF cells require further investigation.

## Conclusion

Our research shows that immune cell infiltration, including Tregs and macrophages, is involved in the pathological process of IVD degeneration. Through single-cell sequencing and clinical experiments, we identified *ID1*, *PTPRK*, and *RAP2C* as hub genes, which may serve as molecular targets for prognostic evaluation and treatment of LDH.

## Data Availability Statement

Publicly available datasets were analyzed in this study. This data can be found here: https://www.ncbi.nlm.nih.gov/geo/query/acc.cgi?acc=GSE154884.

## Ethics Statement

The studies involving human participants were reviewed and approved by The Ethics Committee of the Affiliated Hospital of Chongqing Medical University. The patients/participants provided their written informed consent to participate in this study. Written informed consent was obtained from the individual(s) for the publication of any potentially identifiable images or data included in this article.

## Author Contributions

Conception and design: ZQ. Acquisition of data: LW. Analysis and interpretation of data: LW. Writing, review, and/or revision of the manuscript: JL, JT, and LZ. Study supervision: BW and JL. All authors have read and approved the final manuscript. 

## Conflict of Interest

The authors declare that the research was conducted in the absence of any commercial or financial relationships that could be construed as a potential conflict of interest.

## Abbreviations

IVD, intervertebral disc; NP, nucleus pulposus; AF, annulus fibrosus; DEGs, differentially expressed genes; LDH, lumbar disc herniation; FDR, false discovery rate; NK, natural killer; GO, gene ontology; ELISA, enzyme-linked immunosorbent assay; qRT-PCR, quantitative real-time polymerase chain reaction; t-SNE, t-distributed stochastic neighbor embedding; PLET1, placenta expressed transcript 1; ID1, inhibitor of DNA binding 1.
